# Supplementary material for: Are carbon emissions trading and green financial instruments synergistic? -Comprehensive quantitative research based on content analysis
Source: PLoS One. 2024 Mar 7;19(3):e0298601. doi: 10.1371/journal.pone.0298601 (PMC10919601; doi:10.1371/journal.pone.0298601)
Supplement: S1 Data — (ZIP) [file pone.0298601.s001.zip › Data/地级市Z_F工作报告.zip!/╦─┤¿/┤∩╓▌╩╨/└┤╫╘╣1⁄2╓┌║┼í╛┬φ┐╦╩2╛▌═°í┐_2015.docx]

各位代表：

现在，我代表市人民政府向大会报告工作，请予审查，并请市政协各位委员提出意见。

一、2014年工作回顾

过去一年，是全面深化改革的开局之年，也是我市克难奋进、坚韧前行的一年。在省委、省政府和市委的坚强领导下，在市人大、市政协的监督支持下，我们积极应对复杂严峻形势，始终保持专注发展定力，深入实施“三大总体战略工程”，全市经济保持平稳较快增长，各方面建设取得新的成就。全年实现地区生产总值1347.8亿元，增长8.4%；地方公共财政收入72.4亿元，增长19.1%；全社会固定资产投资1176.2亿元，增长17.3%；社会消费品零售总额560.5亿元，增长13.6%；城镇居民人均可支配收入20939元，农民人均纯收入8945元，分别增长10.7%和11.8%；城镇登记失业率4.0%；人口自然增长率3.8‰；居民消费价格上涨2.1%；节能减排省控目标全面完成。

一年来，我们主要做了以下工作：

——稳增长，经济持续健康发展。全面落实国家和省各项调控政策，及时推出稳增长“十三条”，相继出台市场拓展、金融支持等系列配套措施，从多方面稳定经济增势。突出抓好项目建设，加快实施重点项目150个，集中攻坚重大工程项目25个，重点项目完成投资362亿元。达钢含钒建筑用钢、川投燃气发电、瓮福磷酸盐等一批产业项目陆续投产；达巴铁路、南大梁高速公路和达宣、达开快速通道等一批交通项目加快建设，达营高速公路如期开工，机场迁建进入立项审批，达州交通三年推进方案获批实施；渠江流域综合治理工程项目有序推进，土溪口大型水库项目建议书获国家发改委批复；秦巴医贸园、长德商贸城、二手车市场等一批服务业项目建成营运。城乡消费活力增强，电子商务迅速发展，成功举办第五届秦巴地区商品交易会。全力支持实体经济发展，新增信贷投放146亿元，直接融资规模115亿元，第二期10亿元企业债券获批发行，工业企业应急转贷资金、中小企业贷款风险补偿基金建立运行，土地整理和“双挂钩”项目新增指标2万亩。

——调结构，产业发展加快转型。工业经济提质增效。新培育十亿级企业2户、亿元企业18户，新增规模以上工业企业50户，规模以上工业增加值344.7亿元、增长8.2%。达州经开区成功入选国家循环化改造示范试点园区和全省唯一的首批国家低碳工业试点园区，宣汉、大竹被列为全省首批14个工业强县示范县。完成工业投资344亿元，其中技改投资239亿元。淘汰10户企业落后产能，关闭小煤矿18家，传统资源型和高耗能产业比重下降1个百分点。新增高新技术企业7家，高新技术产业产值达到190亿元。创建市级以上企业技术中心8家、四川名牌产品12个，专利申请突破800件，渠县获批国家知识产权强县工程试点县。服务业快速发展。实现增加值353.2亿元，增长11%，被列为全省服务业发展示范市。红星美凯龙、仁和春天、罗浮新城市广场等城市综合体加快建设，西城达人街、南城阳光耍都等特色街区升级改造如期完成。新增金融机构13家，金融业实现增加值42.5亿元。旅游景区建设取得新进展，成功创建省旅游标准化示范市，实现旅游总收入90.4亿元。现代农业加快推进。粮食生产实现“八连增”。建成农建综合示范区21万亩，新建集中连片特色产业基地16万亩、标准化畜牧养殖小区291个，规范发展家庭农场221家。市级以上龙头企业达到113家，农产品加工收入突破100亿元。三次产业结构由上年的21.4:53.1:25.5调整为20.9：52.9：26.2。

——重建管，城乡面貌明显改观。坚持规划引领，提升“四层架构”，全市城镇化率达到39.4%。强化主城带动，北城滨江、南城三里坪、西城马踏洞和莲花湖新区加快建设，塔坨片区棚户区改造顺利开工，中心城区建成区面积达72平方公里、人口85万。城市综合交通运输体系规划编制完成，残疾人代步车非法营运依法取缔，道路运输“三超”治理和“黑车”载客整治取得实效。中心城区绿化新植改造全面提速，新增城镇绿地38万平方米，全市森林覆盖率达到41.5%。“五治”工程扎实推进，城市管理水平明显提升。县城新区和产业园区加快发展，大竹北城新区、宣汉西区新城、渠县东城新区、开江城普新区、万源东区新城初具规模，7个县（市、区）经济总量均突破100亿元。小城镇加快建设，新增省级试点镇4个，总数达到17个。坚持产村相融建设新农村，新建和改扩建新村（综合体）聚居点337个、幸福美丽新村49个。

——抓改革，发展活力不断增强。推进行政管理体制改革，新一轮政府职能转变和机构改革基本完成；累计取消、调整、下放行政审批事项150项，市级层面核准事项减少50%以上；创新实施“一章”办理，积极推进容缺预审，政务服务效能明显提升。推进工商登记制度改革，新登记企业增长91.1%。推进国资国企改革，市罐头厂、市丝绸厂等4户停产破产企业改革进展顺利，混合所有制经济改革加快实施。推进财税金融体制改革，政府预算体系健全完善，“营改增”试点持续扩大，达州农商行筹建获批，宣汉农商行挂牌运营。推进投融资体制改革，民间投资活力加速释放，民营经济增加值占地区生产总值比重达到59.9%。推进农村改革，农村产权确权登记颁证全面开展，新型农业经营主体快速增加。推进开放型经济发展，东向出川通道达万高速公路全线通车，达州火车站旅客及货运周转量持续增长，达州机场新开通至泉州航线，客货吞吐量分别增长19%、48%；招商引资到位资金659亿元，外贸进出口总额3.2亿美元。教育、科技、文化、医药卫生等领域改革有序推进，全市发展的动力活力得到增强。

——惠民生，人民生活有效改善。各项民生支出达到203.5亿元，占公共财政支出的70.8%，九项民生工程、19件民生实事办理完毕。社会事业加快发展。第一轮学前教育三年行动计划如期完成；新改扩建中小学校121所，城区学校大班额有效化解；西南职教园区加快建设。文化事业繁荣发展，巴山书画院、美术馆、田园大舞台建成开放，市图书馆即将开馆，成功举办第四届全国新农村文化艺术展演和四川省首届农民艺术节暨民间艺术节。实施城乡医疗卫生服务项目257个，引进二级以上民营医疗机构4家，实现医保异地就医即时结算。“单独二孩”政策全面落实。全民健身活动广泛开展。第三次全国经济普查圆满完成。城镇新增就业3.7万人，农村劳动力转移182万人（次）。五项社会保险参保覆盖人数达到496万人次，50.2万城乡低保、5.2万五保对象得到救助安置，15万农村群众实现脱贫。新增（改造）养老床位7100张，新建城乡社区日间照料中心108个。完成棚户区及农村危房改造3.5万户，建设保障性住房5117套。出台了中心城区征地拆迁安置房建设管理办法，加快推进历年累欠150万平方米安置房建设，让拆迁群众早日住进新居。建成通乡通村公路3000公里，完成道路安保工程1680公里。新解决34万农村人口饮水安全问题。加强和创新社会治理，“法律七进”活动深入开展，信访总量持续下降，食品药品和农产品质量安全监管力度加大，安全生产事故起数和死亡人数同比分别下降55%、34%，社会保持和谐稳定。

——转作风，自身建设切实加强。深入开展党的群众路线教育实践活动，扎实推进正风肃纪“9+2+Ⅹ”突出问题集中整治和“庸懒散浮拖”专项治理，行政机关工作作风明显改进。全面贯彻中央八项规定和国务院“约法三章”，对机构编制、办公用房、公务用车进行了清理规范，“三公”经费同比大幅下降。有力推进行政权力依法规范公开运行，在全省率先建成“行政权力公开透明运行系统”。加强行政执法监督，规范自由裁量权“达州经验”全省推广。突出问题导向建章立制，制定出台公共资源交易、政府性投资项目管理等制度规定，依法规范履职能力全面提升。自觉接受市人大及其常委会法律监督、工作监督和市政协民主监督，办结市人大代表建议156件、市政协提案348件。强化行政监察和审计监督，支持执纪执法机关依法履职，严肃查处了一批违纪违法案件。

过去一年，我们扎实推进国防、民兵预备役、人民防空、交通战备、双拥和优抚安置工作，积极做好民族、宗教、外事、侨务、统计、档案、保密、气象、地方志等工作，大力支持工会、共青团、妇联、残联、文联、侨联、科协等开展工作，各方面都取得了新的进步。

各位代表，过去一年的成绩来之不易，这是省委、省政府和市委正确领导、科学决策的结果，是市人大、市政协有效监督、大力支持的结果，是全市人民攻坚克难、共同奋斗的结果。在此，我代表市人民政府，向全市人民，向人大代表、政协委员，向各民主党派、工商联、无党派代表人士和人民团体，向驻达解放军、武警官兵和政法干警，向所有关心支持达州建设发展的社会各界人士表示最衷心的感谢！

在肯定成绩的同时，我们清醒地认识到，达州发展仍面临不少困难和问题。一是经济下行压力较大，投资消费难以实现高增长，部分行业企业生产经营困难。二是产业结构矛盾突出，缺少产业项目和新兴产业支撑，靠引进增量调结构的空间受到制约。三是经济领域改革不少事项还未落地，妨碍发展的体制机制障碍亟待破除，地方债务、金融、房地产等领域潜在风险开始显现。四是基础设施和公共服务领域欠账较多，涉及人民群众切身利益的教育医疗、生态环境、食品药品安全等领域不少突出问题亟待解决。五是为官不为、为官乱为现象仍然存在，依法行政和反腐倡廉建设还需加强。对于这些问题，我们将采取切实有效措施，倾力解决和纠正，绝不辜负全市人民的期望！

二、2015年主要目标任务

2015年，是实施“十二五”规划的收官之年，改革发展任务艰巨繁重。今年政府工作的总体要求是：全面贯彻党的十八大和十八届三中、四中全会精神，按照省委、省政府和市委的总体部署，主动适应经济发展新常态，坚持保位争先、加快发展、追赶跨越工作基调，以提高经济发展质量和效益为中心，深入实施“三大总体战略工程”，着力内需拉动、产业带动、创新驱动，优化投资结构，具化开放合作，狠抓改革攻坚，加强民生保障，强化风险防控，实现经济持续健康发展和社会和谐稳定。

经济社会发展的主要预期目标是：地区生产总值增长8.5%；全社会固定资产投资增长10%；社会消费品零售总额增长12.5%；地方公共财政收入增长9.5%；城镇居民人均可支配收入增长10%，农民人均纯收入增长11%；居民消费价格涨幅控制在3%左右；城镇登记失业率控制在4.1%以内；人口自然增长率控制在5.3‰以内；全面完成省政府下达的节能减排任务。

围绕上述总体要求和预期目标，我们将牢牢把握全局，突出重点，统筹兼顾，在新的起点上全面推进“改革深化年”、“开放合作年”、“项目落实年”、“环境优化年”、“法治建设年”，确保圆满完成全年各项工作任务。

——改革深化年。就是要坚持向改革要速度，向管理要效益，继续深化行政体制、国资国企、财税金融、农业农村、投融资等各领域改革，切实创新城市建设、政府投资、公共财政、国有资产等各方面管理，大力推动理念创新、技术创新、发展方式创新，鼓励改革，大胆创新，先行先试，让市场和社会活力竞相迸发，再添达州发展新动力。

——开放合作年。就是要进一步具化充分开放合作战略，积极实施“走出去”战略，把招商引资作为经济工作的主抓手，扎实开展产业招商、园区招商、驻点招商、委托招商、小分队招商和以商招商活动，继续深化区域合作、银政合作、企地合作、校地合作，举全市之力、集全市之智，强力推动新一轮大开放、大合作、大招商，再拓达州发展新空间。

——项目落实年。就是要着力转变政府工作理念和工作方式，把改革创新、经济发展、城市建设、环境保护、社会事业、民生保障和社会管理等各项政府工作项目化，明确工作的路线图、时间表和责任制，做到工作项目化、项目目标化、目标责任化，抓铁有痕、踏石留印，以过硬作风强执行，以务实态度抓落实，再促达州发展新跨越。

——环境优化年。就是要致力加强“四个环境”建设，持续打好环境治理攻坚战，着力营造更加廉洁高效的政务环境、绿色低碳的生态环境、宜居宜业的人居环境、诚信安全的金融环境，以环境改善凝聚发展共识、集聚人才资源、推动创新创业。通过服务基层、服务企业、服务群众，不断提升政府服务能力和水平，克服疲劳症，提振精气神，再创达州发展新优势。

——法治建设年。就是要始终坚持把推进依法行政摆在政府工作更加突出的位置，牢固树立“法定职责必须为、法无授权不可为”的理念，崇尚法治，厉行法治，切实在法治轨道上履行法定职责、调节社会关系、规范社会行为，着力营造办事依法、遇事找法、解决问题靠法的良好环境，建设法治四川先行区，再续达州发展新篇章。

三、2015年重点工作

（一）坚持内需拉动，保持经济平稳增长

扩大有效投资。继续实施重大项目带动战略，持续推进25个重大工程项目，带动150个重点项目建设，力争完成全社会固定资产投资1300亿元。大力开展“三年交通建设大会战”，确保南大梁高速公路、达巴铁路建成通车，加快达营高速公路和达宣、达开快速通道等项目建设，开工建设巴万高速公路，做好达州机场迁建、渠江航道升级改造、达渝和达万城际铁路等项目前期工作。推进“3+10+1”控制性水利工程建设，加快白岩滩、刘家拱桥、寨子河、土地滩、石峡子5座在建水库工程进度，力争开工建设土溪口、双河口水库。加大产业项目投入，推进先进制造、精细化工、商贸物流、农副产品加工等项目建设，提高工业投资比重，保持服务业投资继续快速增长。实施优质教育、全民健康、百姓安居等民生工程项目，完成投资200亿元以上。抓住国家“十三五”规划和川陕苏区振兴发展规划编制机遇，积极争取特惠政策和项目资金支持，开启下一个五年投资工作新篇章。

促进消费增长。顺应市场个性化、多样化消费需求，扩大教育文化体育、养老健康家政、信息、旅游、住房、绿色等领域消费。大力发展节会经济，积极参加惠民购物全川行、川货全国行等活动，精心办好第六届秦巴地区商品交易会，提高达州产品市场占有率。重视发展电子商务，推进区域电商平台建设，完善农村电商配送网络，鼓励达州名特产品电商化。结合养老保险并轨完善机关事业单位工资制度，适时调整最低工资标准，提高中低收入群众收入，落实带薪休假制度，增强居民消费能力。整顿规范市场秩序，改善消费环境，让群众放心消费。

强化要素保障。全面落实稳增长各项政策措施，更好支持实体经济发展。落实好金融支持政策，发挥工业企业应急转贷资金、中小企业贷款风险补偿基金作用，推动新一轮企业债券发行，支持企业上市融资，新增信贷投放140亿元，直接融资突破120亿元。落实好民营经济发展政策，鼓励社会资本进入特许经营领域，支持达州商会大厦加快建设，力争民间投资占比达到50%以上。落实好财税支持政策，实行结构性减税和普遍性降费，切实减轻企业负担。加快实施土地整理、“双挂钩”项目，新增指标2万亩以上。抓好煤电油气运调度，强化能源资源有效供给。

（二）坚持转型发展，推动产业优化升级

推进新型工业化。全力促进工业转型升级发展，确保规模以上工业增加值增长9%以上。实施产业发展路线图引领计划，培育发展节能环保装备、新能源汽车、新材料、页岩气等高端成长型产业和战略性新兴产业，改造提升冶金、机械、建材、纺织等传统优势产业，确保中石油宣汉净化厂、颜锋铝业等项目竣工投产，开工建设不溶性硫磺、金属装饰保温一体板等项目，加快达钢搬迁改造步伐。具化创新驱动，大力倡导和实施以工业设计为核心的产业设计工程。落实重点工业企业转型升级推进方案，抓好示范工程建设，力争全年技改投入240亿元以上。深入推进产业园区“121”工程，抓好“七通一平”、标准厂房和公共服务平台建设，加快达州经开区创建国家级开发区和大竹、宣汉、渠县工业园区创建省级开发区工作。大力推进企业“百十一”工程，新培育超10亿元企业2户、超亿元企业8户，新增规模以上工业企业50户。推进工业化信息化融合发展，培育信息化示范企业20家。强化企业创新主体地位，培育高新技术企业3家，新增省级以上名牌产品5个，创建市级以上企业技术中心8家，专利申请700件。

提升现代服务业。全面落实服务业支持政策，优先发展五大新兴先导型服务业，推动生产性生活性服务业协同发展，确保服务业增加值增长10.3%以上。充分利用达州区位优势，完善物流体系，促进三次产业联动发展。加快推进经开区铁路专用线建设和火车货站南迁，全面启动秦巴物流园区建设，着力打造区域铁路物流服务基地。扎实抓好杨柳商贸集聚区、复兴商贸物流园、马踏洞金融集聚区等重点项目建设，加快推进红星美凯龙、仁和春天、罗浮新城市广场、升华广场等城市综合体建设，打造“百亿级”服务业集聚区，建设区域服务中心城市。大力促进旅游与文化、农业、康养等产业融合发展，加快百里峡、八台山等优势资源开发和巴人文化考古发掘，启动罗家坝遗址公园建设和城坝遗址发掘工作，提升渠县、大竹、开江等地A级旅游景区品质，培育通川、达川等都市休闲旅游新业态，实施宣汉、万源等为重点的乡村旅游富民工程，办好市第五届旅游发展大会，实现旅游总收入105亿元以上。

发展现代农业。转变农业发展方式，突出农业基础地位，继续实施“一牌三化”和农业“4+8”工程，加快推进“六带三区两基地”建设，繁荣发展农村经济。大力开展粮油高产高效创建活动，粮食总产稳定在284万吨以上。继续抓好现代农业、林业、畜牧业重点县建设，新增特色产业基地10万亩、畜牧养殖小区150个。扶持发展种养大户、家庭农场等新型经营主体，新增市级以上龙头企业14家、标准化农民专业合作社20家、家庭农场50家。加强农业基础设施建设，新建农建综合示范区21万亩，完成病险水库除险加固21座，新增有效灌面8万亩，建成高标准农田14.5万亩。加强农产品质量安全监管，推进农产品精深加工，新获“三品一标”认证10个。实施科普惠农兴村计划，推进市农科所“撤所建院”，提高农业科技服务水平。

（三）坚持改革创新，激发发展动力活力

深化经济体制改革。全力推进国资国企改革，完结市属35户停产破产国企改革，加快39户国企分类改革，做实市投资、达州发展、国资经营三大平台公司，组建市交通投资建设集团公司。改革完善政府预算管理机制，建立健全对政府资金和财政运行全程监管的制度体系；推进专项资金管理改革，规范地方政府债务管理，清理规范税费优惠政策。积极推进金融体制改革，大力发展服务小微企业的中小企业金融机构，加快组建达州农商行、大竹农商行，做大做强达州城商行，构建多层次资本市场体系。深化农村“6+10”专项改革，加快农村“六权同确”登记颁证，建成农村产权交易中心，进一步放活土地经营权。

推进行政体制等领域改革。稳妥推进行政区划调整，加快大竹、渠县撤县设市进程，实施符合条件的乡镇撤乡设镇、撤镇设街道办事处工作。坚持重心下移，强化属地管理，进一步明晰市区体制机制。深化行政审批制度改革，加强政务服务体系建设和公共资源交易管理，把简政放权、放管结合的改革推向深入。深入推进商事制度改革，完善企业注册资本认缴登记制度，建立“宽进严管”的市场主体信用监管体系。加快事业单位分类改革，探索规范公益类事业单位管理。落实户籍制度专项改革方案，有序推进农业转移人口市民化。

（四）坚持开放合作，扩大增量调整结构

深化区域合作。主动对接国家“一带一路”和长江经济带战略，加快构建综合立体交通走廊，积极融入对外开放的大格局，探索建设内陆开放型城市。全面融入成渝发展，加快建设成渝经济区配套产业高地。密切与珠三角、长三角、环渤海等发达地区的联系，积极承接产业转移。加强与川东北和秦巴地区的合作，促进共同发展。推进银政合作、校地合作，深化与央企的合作，争取中、省部门的更多支持。

提升开放实效。着力创新招商引资方式方法，积极参加省内外重大经贸活动，切实加强重点区域、重点产业、重点客商的专题投资促进，持续做好“达商回家”工作。大力实施招大引强战略，继续抓好市级领导挂包10亿元、部门牵头5亿元招商引资项目落地工作，力争引进投资5亿元以上项目50个，招商引资到位资金650亿元以上。鼓励企业充分开拓和利用国际国内两个市场，大力开展对外贸易、工程承包、劳务和技术合作、境外投资，促进结构调整、财富积累和品牌建设。加快达州海关筹建工作，建好达州经开区、大竹工业园区外向型产业基地，力争进出口总额增长10%以上。

（五）坚持主城带动，促进城乡统筹发展

加强中心城市建设。按照新型城镇化和现代化城市发展要求，大力发展城市经济，落实主城带动战略。坚持规划先行和刚性约束，提升完善城市空间、城市景观、城市交通、建筑风貌、重点区域控制规划，开展城市绿地系统、地下综合管廊、地下空间利用、公共服务配套等专项规划编制和州河滨水岸线规划设计。抓好城市规划馆布展工作，确保年内建成开馆。加快环城公路二期、凤凰山隧道、金南大桥等框架性城市道路建设，全面启动城市轨道交通、绕城高速公路前期工作，推动“一城六片”组团交通的迅速转换。坚持扩容提质并重，加快北城滨江、南城三里坪、西城马踏洞和莲花湖、经开区长田坝等城市新区建设，抓好塔坨片区、老城片区、三里坪片区等棚户区改造，着力构建100平方公里、100万人口区域中心城市。

加快县域经济发展。深化扩权强县、扩权强镇改革试点，实施交通、农田水利、城乡环境综合治理三大提升工程，激发县域发展活力，改善县域发展条件。加快县城新区和产业园区建设，拓展县城发展空间，提高产业和人口集聚能力。创新县域经济发展推进机制，加强分类指导，推进宣汉、大竹工业强县示范县建设，抓好渠县、开江现代农业示范县发展，支持万源建设生态旅游示范县，促进县域经济竞相发展、特色发展。继续实施全省“百镇建设行动”，加快建设一批工业型、商贸型、旅游型特色镇，新增省级试点镇4个。

推进城乡协调发展。加大城乡统筹发展力度，推进基础设施向农村延伸、公共服务向农村覆盖。强化农村公路建设和养护，硬油化通乡通村公路1000公里。抓好农村饮水安全工作，让农村居民喝上干净水。推进“宽带乡村”工程，提升农村信息化水平。实施精准扶贫战略，推进五大扶贫工程，落实13条到村到户措施，确保11万贫困对象脱贫。加快幸福美丽新村建设，实施新村建设“1+5”工程和村落改造“5+1”工程，创建一批美丽乡村示范村庄。

（六）坚持环保优先，建设生态宜居达州

继续实施“五治”工程。坚持以打攻坚战的决心，持续推进中心城区环境综合治理，着力加强“智慧城市”建设，提升城市管理满意度。突出抓好背街小巷、城中村、城乡结合部环境卫生治理，规范市场摊点、广告店招、公交站台设置，整治小街小巷200条。全力开展交通治堵，打通滨河路北段一期、凉水井街临通川桥等11条断头路，建设火车站、红旗路、来凤路3座人行天桥。改革公交经营管理体制，组建市公交集团，加快公交首末站场建设，优化公交运行线路设置，提高公交分担率2个百分点。加强城区停车泊位建设管理，新增停车位1万个。规范出租车运营管理，强力整治非法营运行为。加强大气污染防治，抓好城市道路、建筑工地及运渣车辆扬尘治理。积极争创国家卫生城市。

着力推进节能减排。突出抓好节能减排重点工程，加快落后产能淘汰关闭，推进园区循环化改造试点示范，完成“十二五”节能减排目标任务。推进工业、建筑、交通等重点领域节能降耗，加强冶金、建材、化工等重点行业环境污染整治，支持采购绿色环保产品，扩大节能产品运用。加快“气化达州”步伐，以绿色消费促进环境优化。抓好城镇垃圾污水处理设施建设，提升工业危险废物、医疗废物、餐厨垃圾无害化处理能力，开工建设市第二污水处理厂。加强农村环境保护，强化农村面源和畜禽养殖污染治理。严格落实新《环保法》，加大环保执法力度，下决心向污染宣战，使违法排放者付出惨痛的代价。

切实加强生态保护。积极推进天然林保护区、自然保护区和生态功能区建设，扎实抓好新一轮退耕还林工程，完成营造林20万亩，森林覆盖率达到42%以上。大力实施中心城区 “三山两河一湖”生态景观整治，加快城区绿化新植改造，抓好鹿鼎寨、万家河等休闲和湿地公园建设。重视水土保持工作，综合治理水土流失面积100平方公里。严守耕地林地红线，加强建设用地预审管理。强化地质灾害防治，加大生态脆弱区域和灾毁区域生态修复力度。

（七）坚持民生至上，提高人民群众福祉

大力发展社会事业。深化教育综合改革，构建多层次办学体系，满足多样化教育需求。推进义务教育均衡发展，实施义务教育学校标准化建设和农村薄弱学校改造计划，新改扩建中小学校109所。创建达州市青少年儿童科学院。实施第二轮学前教育三年行动计划，加快西南职教园区、达州嘉祥外国语学校建设，推进普通高中教育、职业教育、高等教育和继续教育发展。加强校长教师交流，提升农村学校教育教学水平。抓好市中心医院业务综合楼、市妇幼保健院迁建等重点项目建设，推进县级公立医院综合改革和乡镇卫生院、村卫生室标准化改造，完善分级医疗制度，加强重大传染病防治，提高公共卫生服务能力。加快发展中医药事业。落实利益导向机制，促进人口均衡发展。大力实施文化惠民工程，建成莲花湖演展基地、工青妇活动中心，启动科技馆布展和档案馆建设，抓好县乡村公共文化设施建设和巴文化研究，办好全国第五届新农村文化艺术展演活动和全省第十五届戏曲小品大赛。重视群众体育、竞技体育发展，加强体育基础设施规划建设。倡导社会主义核心价值观，广泛开展家庭美德、职业道德、社会公德创建活动。加强民族、宗教、对台、外事、侨务、统计、档案、保密、气象、地方志等工作。

努力完善保障体系。深入实施更加积极的就业创业政策，支持高校毕业生自主创业，鼓励农民工回乡创业，促进就业困难群体就业，新增城镇就业3.4万人以上。实施机关事业单位养老保险制度改革，完善城乡居民大病保险制度，进一步扩大社会保险覆盖面。抓好城乡低保、五保对象清理规范工作，加大助残、救孤、济困和法律援助等惠民帮扶力度。加快建立社会养老服务体系和发展老年服务产业，新增（改造）养老床位4900张，新建城乡社区日间照料中心110个。大力发展社会福利事业和慈善事业。认真落实“百万安居工程建设行动”，改造农村危房1.5万户，开工建设保障性住房2.2万套、建成9190套。改进住房公积金提取、使用和监督机制。

深入推进依法治理。坚持以法治为引领，创新社会治理方式，推进“平安达州”建设。扎实开展法治示范创建活动，加强全民普法教育。全面落实重大项目、重大决策社会稳定风险评估，完善“大调解”工作体系，依法化解征地拆迁、企业改制、医疗等领域的矛盾纠纷。加强信访工作，切实解决信访突出问题，落实涉法涉诉信访依法终结制度。清理规范社会组织，建立社会组织失信惩戒制度。支持市投融资协会加强行业自律，化解和防范民间金融风险。深化城乡社区治理体制创新，全面推进网格化服务管理。强化社区矫正监管，加强安置帮教工作。加快食品药品及农产品综合质量检验检测中心建设，严厉打击食品药品安全违法行为，确保人民群众“舌尖上的安全”。加强安全生产监管，强化隐患排查整治，推进“打非治违”常态化。强化危机管理，提高突发事件应急处置能力。加大依法管理网络力度。严密防范和依法打击各类违法犯罪活动，确保社会安定有序、人民安居乐业。

推进军民融合发展。切实加强国防教育、国防动员、民兵预备役、人民防空、交通战备、优抚安置工作，支持驻达部队建设，巩固军政军民团结，争创全国双拥模范城市。

四、加强政府自身建设

各位代表，面对改革发展、富民强市的繁重任务，我们将以自我革命的精神，切实加强政府自身建设，着力构建依法规范、高效廉洁、务实为民的政府。

坚持把法治放在心中，全面推进依法行政。坚决维护宪法法律权威，依法规范行使职权，建立完善权力清单、责任清单、负面清单制度。依法科学民主决策，健全法定决策程序，完善政府法律顾问制度和重大决策合法性审查机制。改革行政执法体制，大力推进综合执法，进一步规范执法自由裁量权。加强权力制约监督，完善行政过错责任追究制度，深化政务公开。自觉接受市人大及其常委会的法律监督、工作监督和市政协的民主监督，虚心听取各民主党派、工商联、无党派代表人士和人民团体的意见，重视司法、舆论和公众监督。

坚持把责任放在心中，大力弘扬实干精神。牢固树立进取意识，把握经济新常态，顺应群众新期待，埋头苦干抓落实，推动实现“十二五”规划，精心谋划“十三五”发展。着力巩固党的群众路线教育实践活动成果，持续开展“庸懒散浮拖”专项治理，坚决纠正和反对“四风”。切实改进文风会风，继续精文简会，腾出更多时间深入一线解决实际问题。大力弘扬议大事、抓实事、主动干、敢担当的工作作风，树立保位争先的决心和勇气，不回避矛盾，不畏惧困难，甘于奉献，勇于担当，创造性地推动各项工作落实。

坚持把纪律放在心中，切实加强廉政建设。认真落实从严治党、从严治政各项规定，管住权力、管好钱财，清廉从政、勤俭为民。严格执行中央八项规定和国务院“约法三章”，规范公务接待，推进公车改革，从严控制“三公”经费支出，严禁新建政府性楼堂馆所。健全严格的财政预算、核准和审计制度，加强对重点领域和重点环节的监督，建立健全惩治和预防腐败体系。全面落实党风廉政建设责任制，坚决惩治腐败，以清正廉洁赢得人民群众的信赖。

各位代表，幸福生活需要合力创造，美好未来源自不懈奋斗。让我们紧密团结在以习近平同志为总书记的党中央周围，在省委、省政府和市委的坚强领导下，励精图治，锐意进取，扎实苦干，为加快建设川渝鄂陕结合部区域中心城市和川东北经济区核心增长极而努力奋斗！
